# Dr. Piggot’s Valedictory

**Published:** 1858-06

**Authors:** J. D. Wingate

**Affiliations:** Bellefonte, Pa.


### DR. PIGGOT'S VALEDICTORY.

Not having room for the very excellent Valedictory Address of Dr. Piggot, to the graduating Class of the Baltimore College of Dental Surgery, we cannot refrain from giving place to the following extract—a beautiful eulogy to the memory of the late Dr. Handy.

Though we had not a personal acquaintance with Dr. Handy, we *knew* him, and are assured that so far is Dr. P. from saying too much in these remarks, that he might with great propriety have said much more. Dr. Handy was known and much esteemed by the whole profession. In his death a loss is sustained that will not soon be forgotten.

"But, gentlemen, we have now sufficiently indulged in anticipations of the future. I have said that this is also a season for retrospect. Let us now turn our eyes toward the past. Alas! the 'prospect is not good that way!'

"I look round me and see one vacant chair. Among the accustomed group whom, year after year, I have met upon this platform, I miss one familiar face. The old foe of our race has been among us, and one of our number shall no more be seen among living men. The silent monarch of the tomb has beckoned with his skeleton finger, and our friend has risen and joined that mighty army which in one unbroken procession, daily, hourly, momentarily, marches into the 'silent land.' We cannot let him pass into those shadowy regions without a grateful acknowledgment of his past services, and an honest eulogy upon his many virtues. His long connection with this school, his high standing as a teacher, his well earned reputation throughout the country, make such a recognition eminently proper upon the present occasion.

"My colleagues have selected me to render this last tribute of respect to the memory of our departed friend, and although the choice might have devolved upon one better qualified for the duty, yet in some respects the selection has a certain fitness. Years ago, when I commenced the study of medicine, Dr. Handy gave me my first lesson in practical anatomy; subsequently we became connected as professors in the same medical school, and from that time till the day of his death, I was proud to call him friend, and had the privilege of that intimate association with him which gave me an opportunity of observing his rare qualitities of mind and heart.

"Your late Professor of Anatomy was born in Somerset County, on the Eastern Shore of Maryland, in the year 1812. He completed his preliminary education at the Classical Academy in Newark, Delaware, whence he went to West Point as a cadet. While there he was attacked with a severe indisposition, which totally incapacitated him for undergoing the rigid discipline of that institution, and compelled him to abandon the idea of serving his country in the profession of arms. For some time he was incapable of any exertion, mental or physical. Upon his recovery, he devoted himself assiduously to the study of medicine, and graduated with high distinction at the Washington Medical College of this city. Not long after he had received his diploma he was appointed Demonstrator of Anatomy in that school, and discharged the duties of that position with the zeal and energy that characterized him through life. In 1841 he was elected Professor of Anatomy in this College, and until a few weeks before his death, he continued his lectures. His teachings have enlightened many successive classes of students. You, gentlemen, have had the good fortune to sit under the sound of his voice; and upon you has fallen the great shadow of his loss. I need not, I am sure, recall to your memory his admirable qualities as a lecturer. You all know how faithful, how zealous, how untiring he was in his efforts to impart instruction; how completely he had the interests of the College at heart, how anxiously he labored to elevate the standard of your profession.

"His long association with students led him to perceive more clearly than most men their particular wants. He was convinced that the ordinary method of instruction in Anatomy left much to be desired. He thought that the habit of treating one set of organs by themselves, without reference to others immediately connected with them, was wrong, and tended to confuse the student, and to deprive the study of much of the interest which ought to attach to it. Dr. Handy altered, and, in my opinion, greatly amended the plan of teaching this science. He studied the various organs in their natural relations. In this way the student obtained a comprehensive view of the whole organization of a part; the deep meaning of its minute structure became clear; anatomy was vitalized for him, and assumed the attractiveness of physiology. In 1854 he published a treatise on anatomy, arranged upon this plan, which has become a text-book in all the dental colleges of this country.

Of the character of our deceased friend, it is difficult to speak in terms of moderate eulogy. He was emphatically the "*justum et tenacem propositi virum*" whom Horace praises so highly. It would be difficult to find anywhere a man so rigorously and scrupulously just. In his whole career, not only did never a spot sully the purity of his reputation, but no one who knew him ever dreamed that he could for a moment even design an unjust or dishonorable action. His was that inflexible integrity which has been so lauded among the ancient Romans, and is so rarely found in our degenerate days. His fixed and unalterable determination was always to do right, and his whole life was characterized by a high morality which was incapable of accommodation to circumstances. He never suffered his own convenience or inclinations to interfere with what he considered right.

" In deciding upon any particular line of action, he was extremely cautious. He weighed well all the arguments upon either side of the question, deliberated calmly, and while engaged in determining the point, was always open to conviction; but having once made up his mind, his decision was final and irrevocable. No argument, no entreaty, no threats could move him from his purpose. Intimidation signally failed with him, for that brave heart never quailed before mortal man.

Severe, however, as were his principles, in social intercourse he was the most affable and amiable of men. He was studiously considerate of the feelings of others, and rarely allowed an expression to escape him which could wound the most sensitive nature. Even when roused to honest indignation by marked injustice, he gave expression to his feelings in measured language, and did not suffer the excitement of the occasion to carry his expressions beyond the literal truth. In his intercourse with his brother practitioners, he was not only courteous, but most scrupulous in observing all the little points of medical etiquette. Indeed, tenacious as he naturally was of his own rights, I have known him more than once to sacrifice them rather than incur the suspicion of an inclination to violate those of others. His self-respect led him to demand of others the same even-handed justice which he accorded to them, and if necessary, he did not hesitate to extort it.

"He was also a man of rare purity of heart. Modest as a girl, his long intercourse with the world had left him singularly uncontaminated. In the midst of an active practice, he preserved a chastity of life and character which many a cloistered monk, spending his weary years in mortifying the flesh, sighs for in vain.

"Intellectually, his predominant trait was an ardent love of truth. He subjected all theories to the infallible test of facts, severely analyzed them, and impartially rejected whatever could not bear this *experimentum crucis.* The nature of his mind was such that he demanded the most rigorous demonstration, the most absolute certainty, and could be satisfied with nothing else.

"Upon the particulars of his last illness, I do not propose to dwell. It has too recently passed to enable us to speak of it with composure. You all remember the long anxiety with which you heard of its daily changes; your doubts, your trembling hopes, your foreboding fears; while over him

> 'Triumphant Death his dart
> Shook, but delayed to strike.'

"His illness was, as you all know, a painful one, painful to himself and to his friends, on account of the intellectual darkness which from time to time descended upon him. From the beginning, he himself was assured of its fatal termination; but for him death had no terrors. Across the gathering gloom of the present, his eye of faith saw clearly the everlasting brightness of the future, expanding as he neared it, increasing ever from glory to glory, till the luminous heaven received him, and set the bright barrier of stars between him and us. For us, who have watched his spirit lessening up the blue depths, remains the grief, and the longing and the emptiness of heart—for him, the blessedness of the eternal reward. We sorrow not, therefore, as those without hope, but still our wailings to listen to the voice from heaven, saying, 'Blessed are the dead who die in the Lord.'

This is a simple, safe, and easy method of producing anæsthesia, if it can be thus accomplished. Every thing in this direction that promises any thing should be thoroughly tested. It is desirable to have something with which to accomplish this object, that is safe, efficient, and of easy application.

Let all try the following, and report.—Ed.

[For the Dental News Letter.

ANÆSTHETIC AGENTS.—A SAFE SUBSTITUTE FOR THEM.

Having been somewhat timid in regard to the administration of ether and chloroform, I was desirous to find a safe substitute for them. In the spring of 1855, a young man, standing about seven feet in his boots, one whose appearance would convince you that he was not easily frightened at trifles, presented himself to be relieved of the teeth and stumps of his superior, and also some roots of his inferior maxillary. The operation was scarcely begun, when we both discovered that it was no easy job. He got pale and sick and talked about "backing out." The case promising to be a profitable one, it was very desirable to retain it. I proposed to give him something to allay his nervous excitability, and to stimulate him. The first thing I hit upon was the essence of cloves; I administered about twenty drops and proceeded; found the patient much improved; repeated the dose. Matters proceeded smoothly; repeated it another time; he having now taken sixty drops. He talked like a drunken man, and the operation, though an exceedingly difficult one, was finished without much ceremony. On another occasion I administered thirty drops to a young lady, lanced the gums of her four superior incisors, washed the blood from the lancet and my fingers, and, on turning around, found her head in the same position I left it; I got the forceps quickly, and extracted the four teeth without stopping. On the removal of the last, the blood flowed so profusely that it was with difficulty kept from her clothes; when she was told to spit, she commenced laughing, and wanted to know why her head was fastened while the operation was performed. On being informed that it was not fastened, she seemed rather inclined to doubt it.

P——, a member of Congress, wishing the removal of several "growlers," his gums were so highly inflamed, that it seemed he could scarcely bear to have them touched. Before commencing he went to get some brandy; on returning, thought he had not enough to see him through "so horrid an operation," took ten drops of the essence. When the stumps were out, he pointed to the side of the office, asking whether we saw that fellow run, "How he tears it off." On some persons it appears to have little or no effect. Lately I added to it essence of nutmeg; have not since had much occasion to test the two combined. A few weeks ago a lady took one hundred and fifty drops of the two combined and some brandy, without any apparent effect. This article is cheap, compared with brandy, and is entirely safe. I use less of the oils in preparing the essence than is usually employed. J. D. WINGATE.

Bellefonte, Pa.

